# Modeling Time Requirements of CPS in Wireless Networks

**DOI:** 10.3390/s20071818

**Published:** 2020-03-25

**Authors:** César Huegel Richa, Mateus M. de Lucena, Leonardo Passig Horstmann, José Luis Conradi Hoffmann, Antônio Augusto Fröhlich

**Affiliations:** Software/Hardware Integration Lab, Federal University of Santa Catarina, Florianópolis, SC 88040-900, Brazil; huegel@lisha.ufsc.br (C.H.R.); horstmann@lisha.ufsc.br (L.P.H.); hoffmann@lisha.ufsc.br (J.L.C.H.); guto@lisha.ufsc.br (A.A.F.)

**Keywords:** Cyber-Physical Systems, network schedulability, network scalability, Real-time Constraints, network load analysis

## Abstract

In this paper, we present an approach to assess the schedulability and scalability of Cyber-Physical Systems (CPS) Networks through an algorithm that is capable of estimating the load of the network as its utility grows. Our approach evaluates both the network load and the laxity of messages, considering its current topology and real-time constraints while abstracting environmental specificities. The proposed algorithm also accounts for the network unreliability by applying a margin-of-safety parameter. This approach enables higher utilities as it evaluates the load of the network considering a margin-of-safety that encapsulates phenomena such as collisions and interference, instead of performing a worst-case analysis. Furthermore, we present an evaluation of the proposed algorithm over three representative scenarios showing that the algorithm was able to successfully assess the network capacity as it reaches a higher use.

## 1. Introduction

The combination of digital technologies and physical processes behind contemporary CPS is guided by design methods that take time as a first-order metric [1]. In this realm, timeliness is usually achieved by modeling the embedded systems that support the CPS through a combination of domain decomposition strategies and real-time scheduling [2]: First, functionality is decomposed into simple tasks that run isolatedly on several, independent microcontrollers and interact with each other over a statically modeled interconnect (e.g., ECUs on a CAN bus). Subsequently, classic real-time scheduling algorithms are applied to run multiple tasks on more powerful processors interconnected using off-the-shelf networks (e.g., CIM over Ethernet, automobiles over IEEE 802.11p).

Several researchers [3,4,5,6] addressed the design of CPS assuming a deterministic platform behavior, both in terms of processing and communication, and focusing on specific domains or on specific wireless communication phenomena that might invalidate such an assumption. Analytical models with probabilistic distributions of nodes are used to determine the probability of successful communication [7]. Lee [8] presents an analysis on CPS modeling which emphasizes deterministic models, discussing its limitations and the use of probabilistic models. Network scheduling algorithms are applied on such models taking into consideration the slack time of each message to accommodate network latency [9]. Probabilities can also be applied to order the message flow across the network while considering uncertainties [10]. Nevertheless, the growing acceptance of low-power communication technologies (e.g., Photovoltaic Plant Low-power WSN Monitoring [11]) in the design of CPS (like smart home applications [12]) brings along a pressing demand for ad-hoc interconnects, for which many of these assumptions and models are out of reach or do not correspond to reality. Simulation-based tools can leverage on models that are closely related to the aimed CPS, and therefore yield more realistic results [13]. However, relying on simulations or prototypes to assess scalability and schedulability of CPS may require a considerable effort, especially for complex scenarios.

In this paper, we address the problem of determining whether the demands of a CPS over its interconnect can be matched while considering a time-triggered, convergecast, publish-subscribe network model. We propose an algorithm that can estimate whether a subscription can be accommodated by the system within a margin of safety starting with the most critical ones towards those associated with best-effort tasks.

The main contributions of this work are:

- A Schedulability and Scalability algorithm capable of determining whether a subscription can be handled by a given network topology considering the CPS time constraints;
- The introduction of a network unreliability abstraction factor (modeled as a margin of safety) that impacts the scalability and schedulability analysis by applying a time reservation restraint to the acceptance of subscriptions while enabling the achievement of higher network loads (and therefore use) when compared to the conservative worst-case analysis;
- An Evaluation of the proposed algorithm against simulations considering a wide-range network load with three case studies;
- A discussion about the use of the simulations to adjust the margin of safety to fit the network capacity, thus improving utility.

The remaining of the paper is organized as follows: Section 2 presents the Network Model of our solution. Section 3 covers the proposed algorithm in detail. Section 4 presents the evaluation of the proposed algorithm over three case studies. Section 5 presents a discussion over the algorithm results and applicability to real cases. Section 6 presents related works, comparing them to our approach. Finally, Section 7 presents the conclusions of this paper.

## 2. Network Model

In this section, we describe the network model assumed by the algorithm proposed here in the scope of wirelessly interconnected CPS. We assume a scenario in which network nodes (aka CPS devices, subsystems, or components) interact with a gateway on a convergecast scheme, over an unreliable wireless network. The gateway gathers data from sensor nodes and runs network-wide control algorithms that send commands back to actuator nodes. Nodes can play both roles at once and are supposed to be stationary. We assume the communication protocol used by such nodes and gateways have the following characteristics:

- ***Publish-Subscribe:*** nodes and gateways interact using a publish-subscribe policy, with gateways sending interest messages i(type,region,interval,period,expiry) to express interest on a given type of data, produced in a given region of space, during a given time interval. Nodes matching these criteria periodically send reply messages every period units of time. Data is assumed to be valid from the perspective of applications until they expire at time instant expiry. Response messages r(type,origin,timestamp,expiry,data) carry the requested data along with information about its type, origin, a timestamp, and an expiry (the concept of message expiry is discussed below).
- ***Periodic Behavior:*** all traffic in the network originates from periodic responses to known interest messages. Event-driven applications are not allowed and control messages are either known beforehand and can be accounted for, or are modeled as a reservation of network capacity. The periodic responses respect the Interest period during the Interest time interval.
- ***Expiry:*** data carried by the network is only valid during a given time period, expressed by the expiry of the containing response message (r.expiry). Messages on routing queues are kept ordered by r.expiry, so messages closer to expiration are routed first. Expired messages are discarded.

The role of expires, as well as methods to define them at design-time, have been discussed in [14] and are out of the scope of this paper. However, it is valid to mention that, besides influencing the routing of messages, expires also drive the local scheduling of tasks in network nodes and indirectly define levels of criticality for sets of components in a CPS. Best-effort tasks, for instance, manipulate data with expiries set to infinity, thus seizing network resources only when no other higher criticality subsystems are using them. The complexity of properly defining such expires for data, and consequently for messages, is comparable to that of defining deadlines for tasks but they capture a broader range of characteristics of a CPS in a single concept. Relaxing the specification of expires makes the contributions of this paper applicable to other kinds of networks as well.

## 3. Algorithm

In this section, we introduce an algorithm to assess the scalability and the schedulability of a wireless network transporting time-sensitive data in the context of CPS. The algorithm can decide whether a given set of Interest messages can be handled by a given wireless network modeled as described in the previous section. The network capacity is represented by a parameter ($M_{rate}$) that expresses the maximum MAC transmission rate, considering the duty-cycling and the control message reservation.

Algorithm 1 computes the network load for a set of interests *I* over a set of nodes *N*, the laxity time of each interest, and outputs a structure result, composed of a Boolean (result.accept), a set of the unsupported interests ($result.I_{u}$), and the estimated network load (result.load). The set of Nodes *N* is represented as a connectivity graph *G* = (V.E) with *V* = {v∈V|v is a node in the network.}, and *E* = {(v1,v2)∈E|v1,v2∈V and are able to communicate.}, therefore implicitly defining a static network topology for each execution of the algorithm. Dynamic topologies can still be assessed in terms of scalability and schedulability with multiple executions of the algorithm. If the network can handle *I*, the result.accept will be *true* (the default value, at line 6) and the $result.I_{u}$ will be empty (lines 7). Otherwise, result.accept will be *false* (line 38) and $result.I_{u}$ will contain all the unsupported interests (lines 39).

The network load is estimated by accounting the frequency of each interest *i*. The response frequency is expressed by $R_{rate}$ and calculated for all responding nodes based on the interests period (line 17): ∀n∈N: ∀i∈I {$R_{rate}\leftarrow R_{rate}+1/i.period$|i.region.contains(n)}. The total network load (result.load) is given by result.load = $\left(2*R_{rate}\right)$/$M_{rate}$ (line 36), since for every response message *r*, the node *n* waits for an acknowledgement signal of the next hop (i.e., for all response messages *r* the responding node *n* spends 2 MAC periods, one to send the message and one listening to its acknowledgement). If the resultant load (result.load) is higher than the network capacity (100%), the algorithm signals network overload (result.accept=false) and any new interest on *I* is added to the unsupported interests list $result.I_{u}$ (lines 37 to 40).

Laxity time represents the gateway free time after handling the incoming interests responses with a period less or equal to the current i.period (all the interests already analyzed). A response message *r* has a limit of i.threshold time to arrive at the gateway. The threshold (i.threshold) is the minimum value between i.period and i.expiry (line 9). The interest list *I* ordered ascendingly by the threshold value (line 2), and the node list *N* is ordered descendingly by the number of hops to the gateway (line 3). The time elapsed for an Interest *i* response *r* to arrive at its destination is based on the number of MAC periods spent by the nodes ($n.p_{elapsed}$): the number of periods responding to interests (n.responses), added to the number of periods forwarding messages ($n.p_{rx}$) and the number of waiting periods ($n.p_{wait}$) (lines 23 and 24).

On i.period the node *n* responds to all the interests ii with ii.period lesser or equal to i.period: given an Interest *i*, ∀ii∈I,∀n∈N{n.responses←n.responses+⌊(i.period/ii.period)⌋|ii.period<=i.period&ii.region.contains(n)} (line 15).
**Algorithm 1** Network Load1: **procedure**
Analyze(I, N, MOS, $M_{rate}$, $t_{mac}$)2:  $I^{\prime }\leftarrow I$ ordered by min(i.period,i.expiry) ascendant, where i∈I3: $N^{\prime }\leftarrow N$ ordered by hops(n,sink) descendent, where n∈N4: $R_{rate}\leftarrow 0$5: result.load = 06: result.accept = **true**7: $result.I_{u}\leftarrow \emptyset$8: **for each**
$i\in I^{\prime }$
**do**9:  i.threshold = min(i.period,i.expiry)10:  **for each**
$n\in N^{\prime }$
**do**11:   $n.p_{rx}$ = $n.p_{wait}$ = $n.p_{elapsed}$ = 012:   n.responses = 013:   **for each**
$ii\in I^{\prime }$ | ii.index <= i.index
**do**14:    **if**
n≠sink
**and**
ii.region.contains(n)
**then**15:     n.responses += ⌊(i.period/ii.period)⌋16:     **if**
ii.index = i.index
**then**17:      $R_{rate}$ += (1/i.period)18:     **end if**19:    **end if**20:   **end for**21:  **end for**22:  **for each**
$n\in N^{\prime }$
**do**23:   $n.p_{tx}$ = $n.p_{rx}$ + n.responses24:   $n.p_{elapsed}$ += $n.p_{wait}$ + $n.p_{tx}$25:   **if**
n≠sink
**then**26:    n.next_hop=n.route(sink)27:    $n.next\_hop.p_{rx}$ += n.$p_{tx}$28:    $n.next\_hop.p_{elapsed}$ += $n.p_{elapsed}$29:   **end if**30:   **for each**
$w\in (N_{Rn}-\left\{n.next\_hop\right\})$
**do**31:    $w.p_{wait}$ += $n.p_{tx}$32:   **end for**33:  **end for**34:  $t_{elapsed}$ = $sink.p_{elapsed}*t_{mac}$35:  free = i.threshold - $t_{elapsed}$36:  result.load = $\left(2*R_{rate}\right)$/$M_{rate}$37:  **if** (free/i.threshold) < MOS**or**result.load > 1.0
**then**38:   result.accept = **false**39:   $result.I_{u}\leftarrow result.I_{u}\cup i$40:  **end if**41: **end for**42: **return**
result43: **end procedure**


The number of transmitting periods of a node ($n.p_{tx}$) is n.responses plus the number of periods receiving and forwarding messages ($n.p_{rx}$) (line 23). The number of elapsed periods up to the node *n* on the routing path ($n.p_{elapsed}$) is defined as the sum of the previous nodes elapsed periods (updated at line 28) with the number of periods *n* in transmitting messages ($n.p_{tx}$) and the number of wait periods (periods waiting for neighbors transmission, $n.p_{wait}$) (line 24). The neighbors of a node *n* is the set of nodes on the radio range of *n* (i.e., $\forall n^{\prime }\in N$ $n^{\prime }\in N_{Rn}$ | $n^{\prime }$ is reachable by the Radio of the node n). Finally, as the node list $N^{\prime }$ is ordered descendingly by the number of hops, we update the number of forwarding periods and the number of elapsed periods of $n^{\prime }s$ next hop (lines 25 to 29), and update the waiting time of all of $n^{\prime }s$ neighbors (lines 30 to 32).

With the number of periods elapsed by the nodes during the response of all the interests until *i*, we define the free time (free) on i.threshold by calculating the total time elapsed on the transmissions ($t_{elapsed}$, line 34) as the number of periods of the sink ($s.p_{elapsed}$) times the MAC period ($t_{mac}$) and subtracting this value from the interest threshold (i.threshold) (line 35). The laxity of i.threshold is obtained by dividing free by i.threshold and is compared to the margin of safety MOS. If the laxity is smaller than MOS result.accept is set to false and the interest *i* is added to $result.I_{u}$ (lines 37 to 40).

Wireless networks are unreliable and message transmission is subjected to collision, hidden-node effects, and lossy environments. The impacts of these characteristics over the network are taken into account as MOS. MOS defines a margin of safety for the network transmission capability and is used on the schedulability and scalability analysis of an Interest *i* over the current network usage.

## 4. Case Study

To demonstrate the effectiveness of our approach, we evaluate the Network Load algorithm (Algorithm 1) for a set of CPS scenarios with different Interest sets and compare it to simulation results. We apply the algorithm to a network running Trustful Space-Time Protocol (TSTP) [15].

### 4.1. SmartData and Trustful Space-Time Protocol

The SmartData construct and the Trustful Space-Time Protocol lay the basis to model our experiments. TSTP is an application-oriented, cross-layer communication protocol for CPS. TSTP delivers trusted, timed, geo-referenced, SI-compliant data to applications through the SmartData construct, which promotes a data-centric view of the network.

SmartData encapsulates the data generated by sensors along with metadata to make them self-contained in terms of semantics, spatial location, timing, and security. SmartData also has an expiry as described in Section 2. They are transported by TSTP, which implements the assumed publish-subscribe, periodic network model. Nodes receive interest messages detailing periodicity, expiry and the type of responses. TSTP fits into our described network model in the following ways: i.region is described as a physical location with *x*, *y* and *z* coordinates, SmartData has a type for its data similar to i.type, i.interval is represented by t0 and tf, i.period and i.expiry are defined in the TSTP interest message. Messages are routed to the gateway (sink) on a geographical convergecast strategy, where n.route(sink) returns a forwarder, which is the node that is physically closer to the gateway.

### 4.2. Experimental Setup

**Figure 1:**
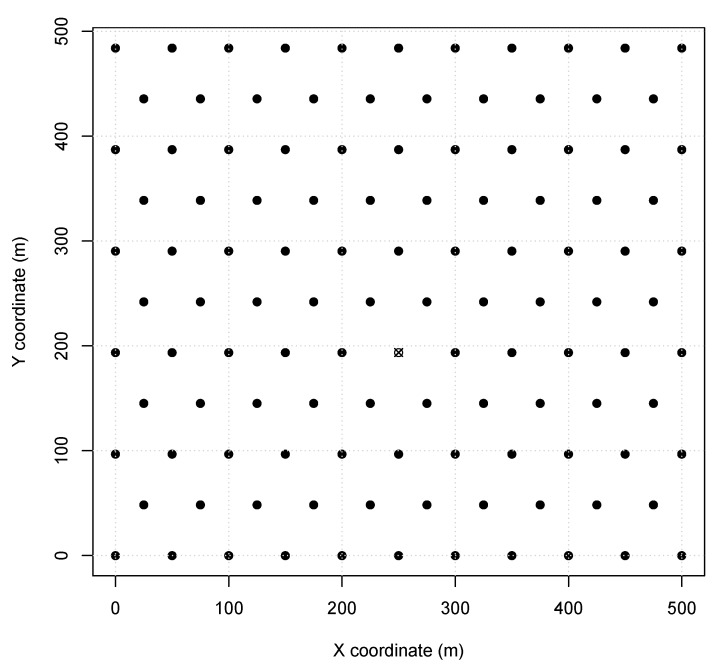
*Scenario 1* node map (sink is X).

The proposed algorithm is evaluated usingCPS scenarios using SmartData and TSTP (see Section 4) with a single central sink according to our Network Model (see Section 2). To validate our algorithm and optimize the parameters, simulations were performed using OMNet++ (version 4.6) with the Castalia framework (version 3.3) to acquire the network delivery ratio. The scenarios represent different aspects of Wireless CPS deployment, such as node distances and placements, based on monitoring environments and industry floor. The scenarios are similar to the ones used in other works [16,17,18]. *Scenario 1* represents a typical monitoring environment, like a solar field monitoring or a soil moisture irrigation system. Nodes in this scenario are configured with TX power of 0dBm (60 m range) and the MAC period of 29.512ms. In this scenario, a 500 $m^{2}$ field is covered with 115 sensor nodes responding to a central sink node. A depiction of *Scenario 1* node placement can be found in Figure 1. Furthermore, all the sensor nodes were instantiated as SmartData responding to the Interest messages described in Table 1, where i.type and i.interval are abstracted for simplification as they are not relevant to the simulation. Also i.period is considered equal to i.expiry.

We evaluate *Scenario 1* in 14 SmartData sets, incremented from $I_{1,1}$ (Interest 1 from *Scenario 1*) to $I_{1,5}$ (i.e., $S_{1}$ = {$I_{1,1}$}, $S_{2}$ = {$I_{1,1}$,$I_{1,2}$}, $S_{3}$ = {$I_{1,1}$,$I_{1,2}$,$I_{1,3}$}, etc.). Moreover, from $S_{5}$ onward the $I_{1,5}$ Interest message is increasingly instantiated, ranging from 10% of the network (11 nodes) on $S_{5}$ to 100% (115 nodes) in $S_{14}$. To assess exactly 10% of the network increasingly at each set, the $I_{1,5}$ instantiation consists of sending a new Interest to each selected node individually. Table 2 summarizes SmartData sets evaluated in this Scenario.

**Figure 2:**
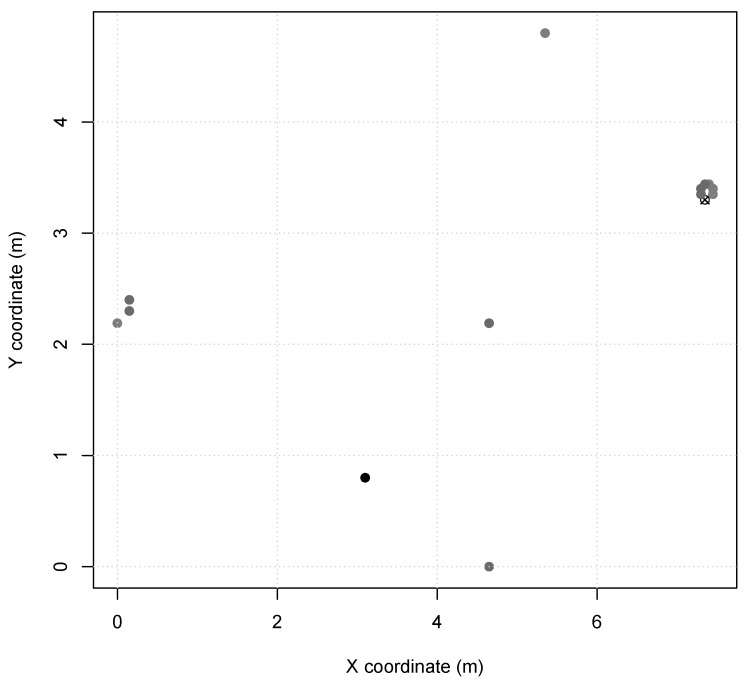
*Scenario 2* node map (sink is X).

**Figure 3:**
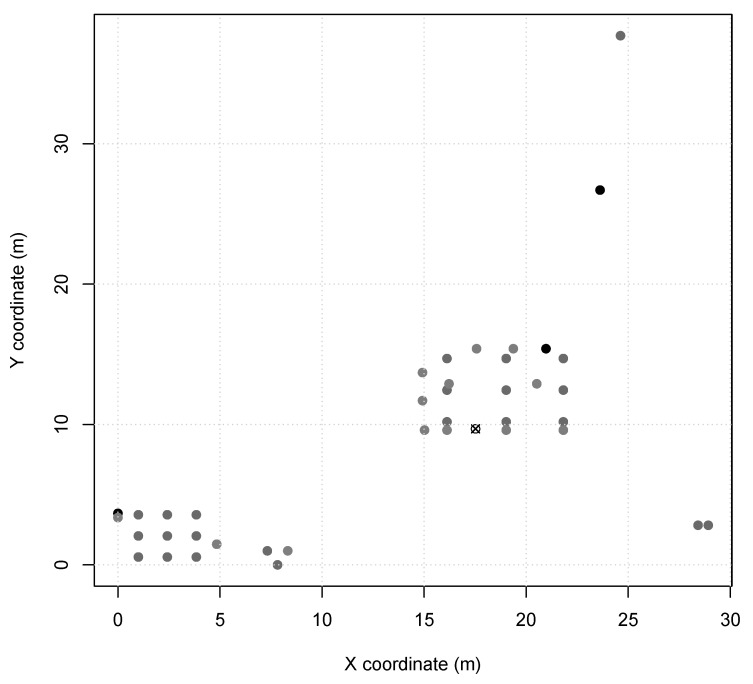
*Scenario 3* node map (sink is X).

*Scenario 2 and 3* represents indoor industry applications, e.g., an autonomous assembly system or a temperature control on a power-plant room. Nodes in these scenarios are configured with TX power of 7dBm (60 m range, same as *Scenario 1*), and the MAC period of 14.288ms in *Scenario 2* and 3.908ms in *Scenario 3*. *Scenario 2* presents node distribution in a small area of 7 m × 5 m (Figure 2), with 13 sensor nodes and 1 sink node. *Scenario 3* presents two node clusters in a larger area of 30 m × 40 m (Figure 3), with 40 sensor nodes and 1 sink node. Furthermore, *Scenarios 2 and 3* nodes are instantiated as SmartData responding to Interest messages as described in Table 3, where i.type and i.interval are abstracted for simplification as they are not relevant to the simulation.

We evaluate *Scenario 2* in 28 SmartData sets, from simulations with a single Interest ($I_{2,1}$) on the network ($S_{1}$) to 15 Interest on the network ($S_{28}$), as summarized in Table 4. SmartData sets until $S_{13}$ are composed of Interests as those described in Table 3. To evaluate the network delivery rate degradation, from $S_{14}$ onward the $I_{2,5}$ Interest message is increasingly instantiated, ranging from 10% (1 node) on $S_{14}$ to 100% (13 nodes) on $S_{23}$, on a rate of 10% per set. As explained in *Scenario 1*, this is done by sending an individual Interest message to each one of the selected nodes. Moreover, this new Interest is again instantiated from $S_{24}$ onward, ranging from 10% of the network on $S_{24}$ (110% in total, 1 node with 2 messages and 12 nodes with 1) to 50% in $S_{28}$ (150% in total, 7 nodes with 2 messages and 5 nodes with 1).

In *Scenario 3*, we evaluate 46 SmartData sets, similar to those of *Scenario 2*, but considering now a greater area and the number of nodes. The SmartData sets go from a single Interest ($I_{3,1}$) on the network ($S_{1}$) to 12 Interests ($S_{46}$), as summarized in Table 5. SmartData sets until $S_{6}$ are composed of $I_{3,1}$ and $I_{3,2}$ instantiations (Table 3). From $S_{7}$ onward, $I_{3,3}$ and $I_{3,4}$ Interests messages are increasingly instantiated, in the same way as the other two already presented scenarios, ranging from 10% (4 nodes) of the network in $S_{7}$ to 100% (40 nodes) in $S_{16}$, on a rate of 10% per set. Moreover, they are instantiated three more times from $S_{17}$ (110% in total, 4 nodes with 2 messages and 36 nodes with 1) to $S_{46}$ (400% in total, 40 nodes with 4 messages), at the same increase cover rate per set.

### 4.3. Results

Each scenario in the previous section was simulated according to the SmartData sets proposed. During simulations, as assumed in Section 2, expired messages are dropped during routing, decreasing the delivery rate metric. Also, one SmartData update is produced at each data period (i.period) in every operational node, providing an equal time to reach the gateway before expiring. The simulations considered a confidence interval of 95%, providing an average of simulation results within the interval.

**Figure 4:**
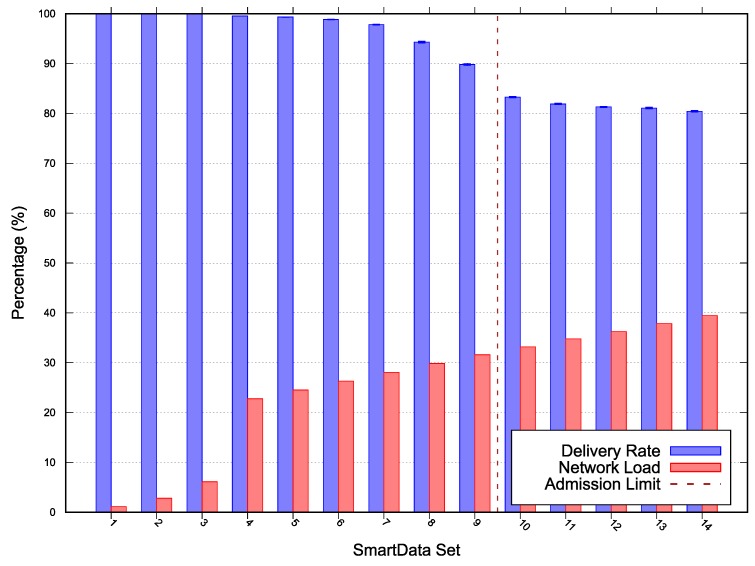
*Scenario 1* simulation and scalability results—Error with 95% Confidence Interval (CI).

**Figure 5:**
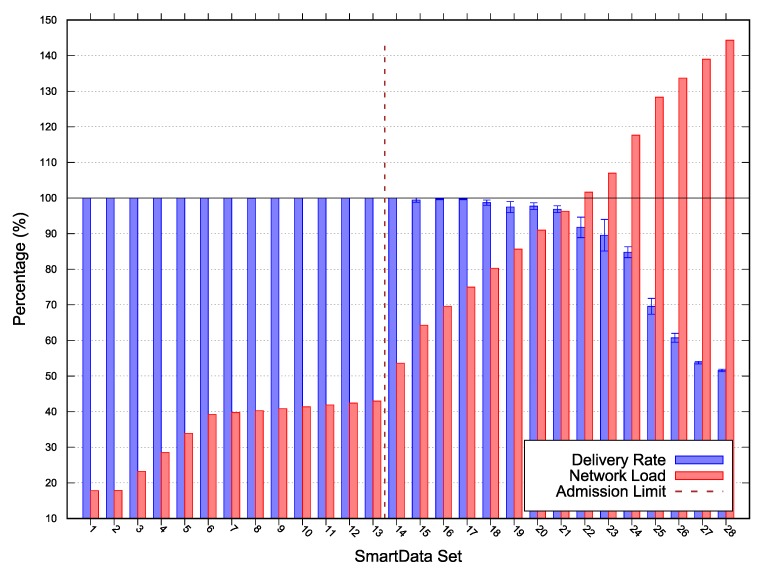
*Scenario 2* simulation and scalability test results—Error with 95% CI.

**Figure 6:**
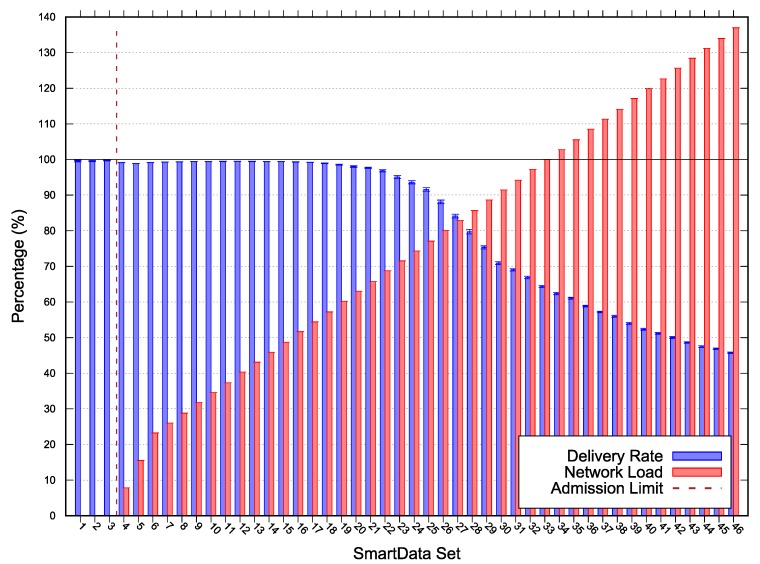
*Scenario 3* simulation and scalability test results—Error with 95% CI.

The results for *Scenarios 1, 2* and *3* depicted in Figure 4, Figure 5, and Figure 6 respectively. The following lines provide a description of each scenario results. A deeper discussion over the possible optimizations, especially for MOS adjustments are presented in Section 5.

In each figure, the vertical line represents the acceptance limit for the Scenario according to Algorithm 1 output (last SmartData set where result.accept = true). In this way we can compare the simulation delivery ratio degradation, represented as blue vertical bars with the proposed algorithm (Algorithm 1) solution. The best-case workloads in terms of channel occupation are represented on the figures as red vertical bars.

For *Scenario 1* the network was configured with a MAC period of 29.512ms, MAC duty-cycling of 42.49ms, no MAC reservation, MAC transmission capacity ($M_{rate}$) of 20.4692 messages/sec, and a MOS of 0. On this evaluation scenario, depicted in Figure 4, the observed algorithm’s acceptance limit was at SmartData set $S_{9}$. The estimated network load was 31.5893% and the minimum laxity time was 3.41006s for the 300s period interest. The algorithm misevaluation is due to simulated network unreliability on on Wireless Sensor Network (WSN) in this topology. Such phenomena are meant to be expressed on the algorithm via the parameter MOS.

In *Scenario 2* we adopted a MAC period of 14.288ms, MAC duty-cycling of 87.765ms, no MAC reservation, MAC transmission capacity ($M_{rate}$) of 37.4841 messages/sec, and a MOS of 0. *Scenario 2* (results depicted in Figure 5) acceptance limit was SmartData set $S_{13}$, with network load of 42.9516%, a minimum laxity time of 0.071392s for a 0.3s period interest. For SmartData set $S_{14}$ the network did also achieved 100% delivered ratio; however, the laxity time was less than MOS (i.e., the estimated free time was less than 0). The presented results corroborate the approach effectiveness since the SmartData set admitted by the algorithm presented simulation with no packet loss.

For *Scenario 3* we used a MAC period of 3.908ms, MAC duty-cycling of 320.878ms, no MAC reservation, MAC transmission capacity ($M_{rate}$) of 86.5202 messages/sec, and a MOS of 0. *Scenario 3* (results depicted in Figure 6) acceptance limit was at SmartData set $S_{3}$. The estimated network load at the admission limit was less than 1%; however, the delivery ratio for the next SmartData set is less than 100% once the elapsed time for the response messages became higher than their expiry.

## 5. Discussion

Algorithm 1 has shown to be effective on *Scenarios 2* and *3*, with its acceptance limit achieved before the delivery ratio decreases from 100%; however the approximation was not precise enough for *Scenario 1* due to the topology definition of such scenario, as in the simulation variability and unreliability were considered and failure in message transmission can affect all the nodes communicating via the failing path. The algorithm’s misevaluation in this scenario could be avoided by adjusting the MOS value (in this case 0). For such adjustment, simulations like the one presented in Section 4 can be used to derive the MOS value from the levels of collision, packet loss and network failure.

*Scenario 1* simulations presented delivery ratios below 100% for every SmartData set after $S_{3}$, which presented the smallest laxity of 33.993%. In this case, MOS can be derived by the smallest value of laxity from the previous Interest Set with 100% delivery ratio, acquiring an approximation of the network constraints. In further experiments, the obtained MOS value will be crucial to delimit the proposed algorithm result acceptance to a closer real-world deployment. On the other hand, when the algorithm acceptance is lower than the presented by simulations results, MOS can be adjusted to achieve a higher network utility. This is the case for *Scenario 2* and *Scenario 3*, where the algorithm acceptance is lower than the network capacity presented by the simulations.

In our study cases, by increasing the Interest message set size we evaluate the schedulability of Interest Message sets for a given network topology, meanwhile evaluating the scalability of the topology when increasing the network traffic. Another possible approach will be to maintain the Interest message set while applying a variation on the network topology. For a network designer, such evaluations are quite useful, mainly to assess the network capacity of the whole network, by setting the topology and Interest message set while varying MOS.

Finally, this work presented an approach to assess periodic real-time CPS wireless networks scalability and schedulability at design-time. The proposed approach models the network variability on its evaluation through a margin of safety MOS to be applied to the laxity on the packet communication. Such parameter can be defined either by a domain specialist or by the simulation results. Our algorithm aims to provide a powerful and flexible tool to estimate and evaluate CPS networks behavior considering domain constraints, able to assess schedulability by increasingly inserting new messages into the network, and scalability by varying network topology.

## 6. Related Works

In this section, we discuss works about network scheduling and network scalability that are related to our proposal, comparing their designs with ours and analyzing the pros and cons.

### 6.1. Schedulability

J. Harbin et al. [19] presents an extension of AirTight [20], a TDMA single-hop protocol, to a multi-hop version with new techniques for slot allocation. The authors assume that nodes are connected to a power supply, to avoid energy-aware techniques (e.g., MAC duty-cycling). To schedule the transactions of conflicting nodes (inside a collision domain), a topology graph is derived containing connection and interference data assisting the division of the channel into time slots. Additionally, each node has a set of local FIFO queues to schedule messages by fault tolerance criticality. The authors initially divide the time slots between the conflicting nodes based on their use. In our approach, we assume a periodic-behavior for the messages exchanged over the network, in contrast to the hybrid model (periodic plus event-driven) they assume. The acceptance of unbound event-driven traffic can easily place a system outside the scope of traditional CPSs and therefore we avoided it. Nevertheless, setting the expiry of an event-driven message to infinity in our scheme causes that message to be routed without interfering on periodic traffic. Both approaches take into consideration the interference of non-deterministic behavior caused by known phenomena of wireless networks. Our algorithm relies on the MOS parameter to abstract the variations in latency caused by interference, while their protocol assumes a fault model applied at each hop. Their approach is certainly more precise, since the fault model can be adjusted to mimic conditions that are specific to individual network nodes. However, the burden of defining such models can render the modeling of the network as a whole far more complex and hence can compromise its usability as a design tool.

The mixed criticality they propose for local scheduling at each node can be abstracted in our approach within each interest i.expiry parameter prioritizing the shorter expires through all the network in only one attribute. As mentioned, the usage of i.expiry can even be extended to define a best-effort class of messages. The authors also claim that the mixed-criticality presented can handle both event-driven and time-triggered traffic. However, within a static slot-table allocation, event-driven messages must be taken into account beforehand and can compromise time-triggered ones (a dynamic reallocation of slots is mentioned by the authors as a future work).

Nayak et al. [21] introduces Time-Sensitive Software-Defined Networks (TSSDN), providing real-time guarantees for time-triggered traffic in time-sensitive systems. The paper proposes a network controller with a global view of the time-triggered packets and the network topology, computing the routes and transmission schedule. The proposed network controller does not take into account network latency variations as it uses a static scheduling scheme with strict time-slot allocations. In our algorithm, the MOS parameter is used to model network latency variations. Moreover, each node accounts for its neighbors’ transmissions, thus avoiding the necessity of a time-slot allocation for the schedulability analysis. The presented network controller classifies messages in two groups, time-triggered or best-effort, limiting criticality representativeness. On the other hand, our algorithm indirectly establishes priorities through expiries, enabling a smooth multi-priority scheme. Our algorithm also returns a priority-sorted list of the lowest-priority rejected interests in a case of saturation.

Ting et al. [22] present a capacity planning tool for high and sustainable bandwidth non-periodic networks that provide 7 different strategies for analyzing the load of the network (e.g., length, latency, and weight). The authors defined a representation model of network topology and traffic through matrices. The algorithm evaluates capacity by calculating the traffic between nodes according to the traffic and topology matrices, composed of an end-to-end approximated traffic. The proposed routing strategies provide different load distribution through the network considering disjoint paths. The length metric routes the traffic through the shortest path from source to destination (similar to TSTP), the latency metric routes the traffic through the lowest latency path (a latency matrix is necessary for this strategy), and the weight metric balances the traffic between the paths based on previous allocations. The presented tool, in contrast to our algorithm, does not provide an evaluation directly based on a set of messages, requiring the user to acquire traffic information through simulations and then convert it to a traffic matrix. Moreover, the network model is not necessarily convergecast nor accounts for real-time and periodic behavior constraints. Thus, no priority is assigned on the traffic abstraction, as the tool output is focused on how much traffic each node can handle. Our approach differs in this respect by considering a more constrained environment, with real-time requirements and periodic behavior, while accounting for wireless network unreliability (i.e., MOS) on the schedulability test.

### 6.2. Scalability

Gopalakrishnan Iyer et al. [7] proposes an analytic model for smart utility resources measuring network (e.g., gas, water and electricity) that scales as a function of link reliability, demonstrating a correlation between the network size and the maximum expectation of packet transmission success. The node placement follows a Poisson spatial distribution model, as the authors note, with a collector (i.e., gateway), placed at the center, and nodes placed according to the distribution based on transmission characteristics (e.g., transmission power and range), network range and a density parameter (i.e., ${\left(transmission\;range/network\;radius\right)}^{2}$), and assuming all nodes have the same transmission range. The scalability analysis is done over the defined network topology, the nodes transmission range, the network radius and the probability of connection success between hops to find the necessary amount of nodes to achieve the maximum expectation packet transmission success. However, the accuracy of the proposed analytical model is highly dependent on the network topology following the statistical placement, as the node density is a key element to its correctness. Real-world scenarios have obstacles and other limitations on nodes’ placement, thus affecting the model accuracy. Our approach can evaluate scalability of convergecast multi-hop networks without limiting network topology. They propose increasing network density (i.e., number of nodes) as a solution for increasing message delivery probability on faulty scenarios. Nevertheless, the hidden-node phenomena worsen along with the number of routes transmitting the same message on a convergecast multi-hop network, possibly affecting the packet transmission delivery success. Furthermore, a scalability analysis for the maximum expectation of packet transmission success is not a guarantee of timeliness in critical scenarios, as a lower bound of packet transmission success is not presented. To account for critical scenarios, our approach uses the demand to evaluate the scalability of a network.

Zats et al. [23] demonstrate the ability to scale a scheduling approach for Time-Synchronized Channel Hopping (TSCH) networks, such as WirelessHART, consisting of 10,000 nodes within a 0.1 Km${}^{2}$, and a response period of 10 s. Aiming at a collision-free operation, the scheduling algorithm uses 3X provisioning factor and spatially reuses the superframe cells according to the average number of hops since the links simultaneously transmitting are not on the radio range one from another. Our approach differs by adding a new level of configuration to our algorithm, where the nodes do not necessarily need to be configured with the same sampling rate. Moreover, we do not use slot allocation, since it is a time-consuming task when considering multi-hop convergecast scenarios. Also, their approach focuses on demonstrating whether a TSCH network is scalable in a specific scenario, while our algorithm focuses on assessing the scalability and schedulability of a given network deployment and a given set of Interests.

Agamy et al. [24] defines a model to analyze the performance of WSN with N-BURST traffic model that allows us to analytically investigate the impacts of bursty traffic on the mean packet delay and buffer overflow probability. The WSN model assumes, however, that all nodes are able to directly communicate with the sink, limiting the analysis to a single-hop network, and do not consider real-time constraints. Our model differs by assuming a multi-hop network in the schedulability and scalability analysis and by considering priority and real-time constraints. The experiments applied variations to the distribution of the ON-times to estimate the mean Package Delay and the buffer overflow probability, while our algorithm estimates the network load and our experiments measure the delivery ratio for simulations that have a constant distribution of burst periods.

While not directly related to scheduling or scalability Younis et al. [25] presents an analysis of the importance of node placement for WSN, stating it has fundamental importance, affecting the whole network performance, since it affects the ability of the network to correctly sense an event, and also the number of possible disjoint paths towards the sink. The node placement directly affects the parameters of our proposed algorithm in Section 3.

## 7. Conclusions

In this paper, we have presented an algorithm capable of determining whether a subscription can be accommodated by the system within a margin of safety, while considering a time-triggered, convergecast, publish-subscribe network model. The proposed approach enables the achievement of higher loads by analyzing the network with a new perspective, in which the acceptance limits are given by a margin-of-safety over the messages deadlines instead of the worst-case. The algorithm uses a given set of nodes and their implicit topology, a given set of interests, a MAC rate and a margin of safety to decide whether the current configuration of the network is schedulable when responding to the given interest set. The algorithm was evaluated through simulation, comparing the simulated delivery rate to the algorithm acceptance for a given interest set in each of the three scenarios. Figure 4, Figure 5 and Figure 6 depict an overview of the simulations and the algorithm results. The algorithm successfully identifies each scenario capacity, except on *Scenario 1*, due to the network topology and the MOS underestimation. Moreover, we discussed over MOS value adjustment according to the simulation results, fitting the network specification by reducing the acceptance limit or increasing utility corroborating the approach’s effectiveness.

## Figures and Tables

**Table 1:** *Scenario 1* evaluation Interest messages.

*Scenario 1*	
Interest	*selected nodes/period*
$I_{1,1}$	115 nodes w/900s
$I_{1,2}$	115 nodes w/600s
$I_{1,3}$	115 nodes w/300s
$I_{1,4}$	115 nodes w/60s
$I_{1,5}$	[11..115] nodes w/60s

**Table 2:** *Scenario 1* evaluation SmartData sets.

Set	Period	Set	Period
$S_{1}$	900 s	$S_{8}$	0.4×60 s, $S_{4}$
$S_{2}$	600 s, $S_{1}$	$S_{9}$	0.5×60 s, $S_{4}$
$S_{3}$	300 s, $S_{2}$	$S_{10}$	0.6×60 s, $S_{4}$
$S_{4}$	60 s, $S_{3}$	$S_{11}$	0.7×60 s, $S_{4}$
$S_{5}$	0.1×60 s, $S_{4}$	$S_{12}$	0.8×60 s, $S_{4}$
$S_{6}$	0.2×60 s, $S_{4}$	$S_{13}$	0.9×60 s, $S_{4}$
$S_{7}$	0.3×60 s, $S_{4}$	$S_{14}$	1.0×60 s, $S_{4}$

**Table 3:** *Scenarios 2 and 3* Interest message base configurations.

*Scenario 2*		Scenario 3	
Interest	*selected nodes/period*	Interest	*selected nodes/period*
$I_{2,1}$	1 node w/60 s/0.3 s	$I_{3,1}$	3 nodes w/60 s/0.3 s
$I_{2,2}$	1 node w/0.3 s/0.3 s	$I_{3,2}$	3 nodes w/0.3 s/0.3 s
$I_{2,3}$	4 nodes w/1 s/1 s	$I_{3,3}$	[4..40] nodes w/1 s/1 s
$I_{2,4}$	7 nodes w/10 s/10 s	$I_{3,4}$	[4..40] nodes w/10 s/10 s
$I_{2,5}$	[1..13] nodes w/1 s/1 s	-	-

**Table 4:** *Scenario 2* evaluation SmartData set.

Set	Period	Set	Period
$S_{1}$	0.3 s	$S_{15}$	0.2×{1 s}, $S_{13}$
$S_{2}$	60 s, $S_{1}$	$S_{16}$	0.3×{1 s}, $S_{13}$
$S_{3}$	60 s, $S_{2}$	$S_{17}$	0.4×{1 s}, $S_{13}$
$S_{4}$	1 s, $S_{3}$	$S_{18}$	0.5×{1 s}, $S_{13}$
$S_{5}$	1 s, $S_{4}$	$S_{19}$	0.6×{1 s}, $S_{13}$
$S_{6}$	1 s, $S_{5}$	$S_{20}$	0.7×{1 s}, $S_{13}$
$S_{7}$	10 s, $S_{6}$	$S_{21}$	0.8×{1 s}, $S_{13}$
$S_{8}$	10 s, $S_{7}$	$S_{22}$	0.9×{1 s}, $S_{13}$
$S_{9}$	10 s, $S_{8}$	$S_{23}$	1.0×{1 s}, $S_{13}$
$S_{10}$	10 s, $S_{9}$	$S_{24}$	1.1×{1 s}, $S_{13}$
$S_{11}$	10 s, $S_{10}$	$S_{25}$	1.2×{1 s}, $S_{13}$
$S_{12}$	10 s, $S_{11}$	$S_{26}$	1.3×{1 s}, $S_{13}$
$S_{13}$	10 s, $S_{12}$	$S_{27}$	1.4×{1 s} $S_{13}$
$S_{14}$	0.1×{1 s}, $S_{13}$	$S_{28}$	1.5×{1 s}, $S_{13}$

**Table 5:** *Scenario 3* evaluation SmartData sets.

Set	Period	Set	Period	Set	Period
$S_{1}$	60 s	$S_{17}$	0.3 s, 1.1×{1 s, 10 s}, $S_{6}$	$S_{33}$	0.3 s, 2.7×{1 s, 10 s}, $S_{6}$
$S_{2}$	60 s, $S_{1}$	$S_{18}$	0.3 s, 1.2×{1 s, 10 s}, $S_{6}$	$S_{34}$	0.3 s, 2.8×{1 s, 10 s}, $S_{6}$
$S_{3}$	60 s, $S_{2}$	$S_{19}$	0.3 s, 1.3×{1 s, 10 s}, $S_{6}$	$S_{35}$	0.3 s, 2.9×{1 s, 10 s}, $S_{6}$
$S_{4}$	0.3 s, $S_{3}$	$S_{20}$	0.3 s, 1.4×{1 s, 10 s}, $S_{6}$	$S_{36}$	0.3 s, 3.0×{1 s, 10 s}, $S_{6}$
$S_{5}$	0.3 s, $S_{4}$	$S_{21}$	0.3 s, 1.5×{1 s, 10 s}, $S_{6}$	$S_{37}$	0.3 s, 3.1×{1 s, 10 s}, $S_{6}$
$S_{6}$	0.3 s, $S_{5}$	$S_{22}$	0.3 s, 1.6×{1 s, 10 s}, $S_{6}$	$S_{38}$	0.3 s, 3.2×{1 s, 10 s}, $S_{6}$
$S_{7}$	0.3 s, 0.1×{1s, 10 s}, $S_{6}$	$S_{23}$	0.3 s, 1.7×{1 s, 10 s}, $S_{6}$	$S_{39}$	0.3 s, 3.3×{1s, 10 s}, $S_{6}$
$S_{8}$	0.3 s, 0.2×{1s, 10 s}, $S_{6}$	$S_{24}$	0.3 s, 1.8×{1 s, 10 s}, $S_{6}$	$S_{40}$	0.3 s, 3.4×{1s, 10 s}, $S_{6}$
$S_{9}$	0.3 s, 0.3×{1s, 10 s}, $S_{6}$	$S_{25}$	0.3 s, 1.9×{1 s, 10 s}, $S_{6}$	$S_{41}$	0.3 s, 3.5×{1s, 10 s}, $S_{6}$
$S_{10}$	0.3 s, 0.4×{1s, 10 s}, $S_{6}$	$S_{26}$	0.3 s, 2.0×{1 s, 10 s}, $S_{6}$	$S_{42}$	0.3 s, 3.6×{1s, 10 s}, $S_{6}$
$S_{11}$	0.3 s, 0.5×{1s, 10 s}, $S_{6}$	$S_{27}$	0.3 s, 2.1×{1 s, 10 s}, $S_{6}$	$S_{43}$	0.3 s, 3.7×{1s, 10 s}, $S_{6}$
$S_{12}$	0.3 s, 0.6×{1s, 10 s}, $S_{6}$	$S_{28}$	0.3 s, 2.2×{1 s, 10 s}, $S_{6}$	$S_{44}$	0.3 s, 3.8×{1s, 10 s}, $S_{6}$
$S_{13}$	0.3 s, 0.7×{1s, 10 s}, $S_{6}$	$S_{29}$	0.3 s, 2.3×{1 s, 10 s}, $S_{6}$	$S_{45}$	0.3 s, 3.9×{1s, 10 s}, $S_{6}$
$S_{14}$	0.3 s, 0.8×{1s, 10 s}, $S_{6}$	$S_{30}$	0.3 s, 2.4×{1 s, 10 s}, $S_{6}$	$S_{46}$	0.3 s, 4.0×{1s, 10 s}, $S_{6}$
$S_{15}$	0.3 s, 0.9×{1s, 10 s}, $S_{6}$	$S_{31}$	0.3 s, 2.5×{1 s, 10 s}, $S_{6}$	-	-
$S_{16}$	0.3 s, 1.0×{1s, 10 s}, $S_{6}$	$S_{32}$	0.3 s, 2.6×{1 s, 10 s}, $S_{6}$	-	-
